# Definitive radiotherapy for early (T1-T2) Glottic Squamous cell carcinoma: a 20 year Cleveland clinic experience

**DOI:** 10.1186/1748-717X-7-193

**Published:** 2012-11-19

**Authors:** Mohammad K Khan, Shlomo A Koyfman, Grant K Hunter, Chandana A Reddy, Jerrold P Saxton

**Affiliations:** 1 Winship Cancer Center, Emory University, Atlanta, GA, USA; 2 Taussig Cancer Institute, Cleveland Clinic, Cleveland, OH, USA

**Keywords:** Glottic carcinoma, Larynx, Outcome, Radiotherapy, Squamous cell, Carcinoma

## Abstract

**Purpose:**

To report our 20 yr experience of definitive radiotherapy for early glottic squamous cell carcinoma (SCC).

**Methods and materials:**

Radiation records of 141 patients were retrospectively evaluated for patient, tumor, and treatment characteristics. Cox proportional hazard models were used to perform univariate (UVA) and multivariate analyses (MVA). Cause specific survival (CSS) and overall survival (OS) were plotted using cumulative incidence and Kaplan-Meir curves, respectively.

**Results:**

Of the 91% patients that presented with impaired voice, 73% noted significant improvement. Chronic laryngeal edema and dysphagia were noted in 18% and 7%, respectively. The five year LC was 94% (T1a), 83% (T1b), 87% (T2a), 65% (T2b); the ten year LC was 89% (T1a), 83% (T1b), 87% (T2a), and 53% (T2b). The cumulative incidence of death due to larynx cancer at 10 yrs was 5.5%, respectively. On MVA, T-stage, heavy alcohol consumption during treatment, and used of weighted fields were predictive for poor outcome (p < 0.05). The five year CSS and OS was 95.9% and 76.8%, respectively.

**Conclusions:**

Definitive radiotherapy provides excellent LC and CSS for early glottis carcinoma, with excellent voice preservation and minimal long term toxicity. Alternative management strategies should be pursued for T2b glottis carcinomas.

## Introduction

Several institutions have reported long term outcomes of patients with T1-2N0 SCC of the glottis treated with definitive radiotherapy[1-10]. The five-year local control (LC) rates have ranged from 82-94% for T1a, 80-93% for T1b, 62-94% for T2a, and 23-73% for T2b. We report our first 20 year institutional outcome, and identify patient, tumor, and treatment related factors associated with inferior outcomes.

## Methods and materials

We obtained institutional review board (IRB) approval to retrospectively review the charts of all patients treated with definitive radiotherapy at the Cleveland Clinic between 1986–2006. All patients had biopsy-proven invasive SCC of the glottis, staged T1 or T2 with negative lymph node disease, and had received an uninterrupted course of radiotherapy. Patients were excluded if they previously had major surgery of the neck or the glottis, had a synchronous primary, or had received chemotherapy. Minor surgery (stripping for squamous cell carcinoma in-situ (SCIS) or minor cordotomy) was allowed. The AJCC 6^th^ edition [11] was used to stage all patients, but with further sub-classification of T2 patients. Patients were staged as follows: T1 included tumor confined to a single vocal cord (T1a) or both vocal cords (T1b) with normal vocal cord mobility; T2 included tumor with supra- or subglottic extension and further subdivided into T2a (without) or T2b (with) impaired vocal cord mobility.

**Figure 1:**
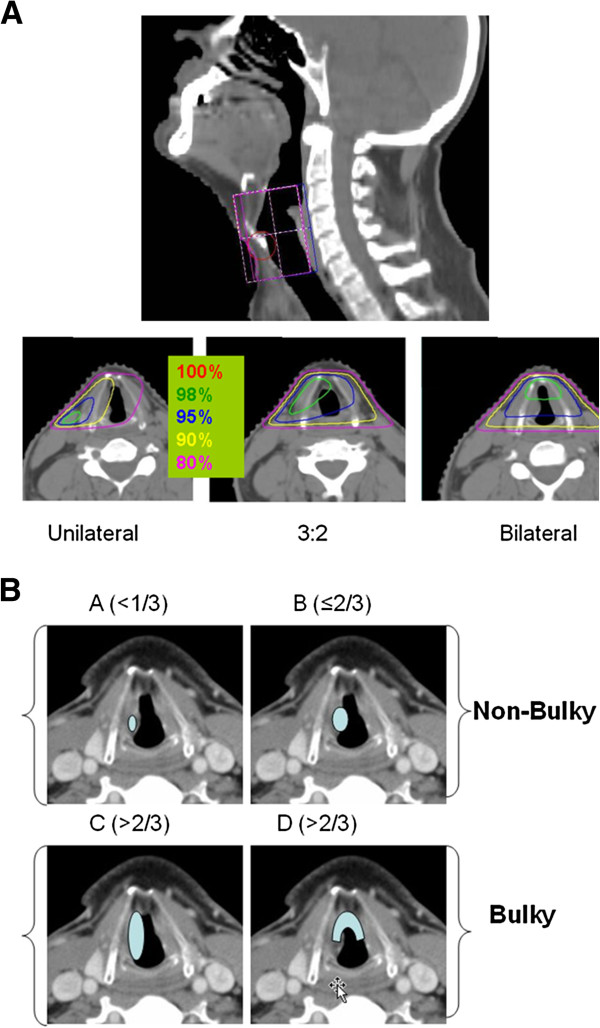
Example of Arrangement & Weighting of Fields (1A) and Comparison of Non- Bulky vs. Bulky Tumor (1B).

All patients were treated with radiotherapy alone using either a unilateral field or a weighted opposed lateral field technique (Figure 1A). Standard field borders were used in most cases for both techniques: 1) superior: mid thyroid notch; 2) inferior: bottom of cricoid cartilage; 3) posterior: 1 cm posterior to the thyroid cartilage but anterior to the vertebral body; 4) anterior: 1 cm anterior to the skin of the neck (“flash”). Most patients were treated using 5500 cGy (range 4400–6940 cGy) in 25 fractions of 220 cGy per fraction (range 180–225 cGy) using a larger field followed by a boost to a smaller volume “cone down” of 1320 cGy (range 600–2520 cGy) using 220 cGy per fraction (range 180–225 cGy) for a total dose of 6820 cGy (range 6300–7264) to the tumor. Higher radiation doses were reserved for patients with more bulky T2 tumors. Most patients underwent a cone down after 5500cGy where the posterior border was placed immediately posterior to the arytenoids, unless tumor extended to this region. Unilateral fields were used for unilateral and well localized tumors. Bilateral fields were used for all other tumors using one of three techniques depending on the relative distribution of the tumor across both vocal cords: equally weighted, weighted 2:1, or weighted 3:2. The neck nodes were treated only in cases where there was significant supragottic or sublgottic extension suggesting increased likelihood for subclinical nodal involvement. This methodology has been kept consistent over the 20 yrs of this study, and is part of the routine practice at our institution.

All statistical computations were performed using SAS version 9.2 (SAS Institute Inc., Cary, NC software. Local control (LC) and overall survival (OS) were plotted using the Kaplan-Meier method. The log-rank test was used to detect statistically significant differences among survival curves. Cause specific survival (CSS) was calculated using the cumulative incidence method. UVA and MVA analyses were performed via Cox regression analysis for the endpoint LC. The following parameters were included in the UVA: age, gender, race, smoking status, heavy alcohol consumption, tumor bulk (amount of cord involved), grade, histology, T-stage (T2 vs T1 and T2b vs all T1/T2a), anterior commissure involvement, supraglottic/subglottic extension, daily dose ≤2 Gy, total dose ≤66 Gy, field weighting (unilateral vs. bilateral equally weighted vs. bilateral unequally weighted), and total treatment time. Tumor bulk was modeled as a continuous variable and classified as involvement of > 2/3 of a cord involved by tumor (Figure 1B). Anterior commissure involvement with extension beyond 1/3 of each cord was categorized as a bulky tumor (Figure 1B). This classification of tumor bulk is identical to that published by Reddy et al. [12] Total dose and treatment time were also modeled as continuous variables. Factors that were significant (p-value <0.05) in the UVA were then included in a MVA. Quality of life variables that were assessed to determine acute and late toxicity post radiation included: patient and physician reported voice quality pre- and post treatment, physician reported laryngeal edema as noted on direct laryngoscopy, patient reported symptomatic dysphagia requiring dilatations, pre and post treatment trismus, the need for salvage surgeries, and the patterns of failure.

## Results

Patient, tumor and treatment related characteristics are depicted in Table 1, along with their association with local control on UVA. The mean follow-up time was 5.6 years. The mean age of the patients was 67 years. The median dose was 6820 cGy delivered at 212 cGy per fraction in 32 fractions over 45 days. On UVA, heavy alcohol use during radiation (p = .02), anterior commissure involvement (p = .05), stage (p = .02), total radiation dose (p= 0.04), subglottic extension (p = .05), and field weighting (p =0.01) were predictive for poor local control. On MVA, concurrent heavy alcohol usage (p < 0.01), stage T2b (p=.02), and field weighting (p = .025) were significant for poor local control (Table 2). Unilateral fields (p<0.03) and equally-weighted bilateral fields (p <0.04) were associated with improved local control compared to bilateral unequally weighted fields. Bilateral equally weighted fields and unilateral fields were equally effective (p = 0.95).

**Table 1:** Patient (n – 141) Characteristics and Univariate Analysis for Impact on Local Control

**Characteristic**	**# of Patients (range or %)**	**p value**
Mean Age (yrs) at end of radiation	67 (33–94)	0.9055
Gender		0.3902
male	125 (88.7%)	
female	16 (11.3%)	
Race		0.1133
Caucasian	119 (84.4%)	
Non-caucasian	22 (15.6%)	
Median Pack Years	44 (0–135)	0.6769
Smoker		0.3530
Never	12 (8.5%)	
Former	92 (65.2%)	
Current	32 (22.7%)	0.5568
Unknown	5 (3.5%)	
Heavy Alchohol Use		0.0630
Never (Reference)	89 (63.1%)	
Former	21 (14.9%)	0.8836
Current	19 (13.5%)	**0.0222**
Unknown	12 (8.5%)	
Ant Commissure		**0.0505**
Involved	71 (50.3%)	
Non Involved	52 (36.9%)	
Unknown	18 (12.8%)	
Tumor Bulk		0.6386
Bulky	55 ( 39%)	
Non bulky	86 ( 61%)	
Hemoglobin (g/dL)	14.2	
Grade		0.6614
Well (Reference)	43 (30.5%)	
Moderate	57 (40.4%)	0.7192
Poor	14 (10%)	0.3646
Unknown	27 (19.1%)	
Stage (T2b vs T1a/b/2a)		**0.0205**
1a	65 ( 46%)	**0.0033**
1b	21 (14.9%)	0.1766
2a	29 (20.6%)	0.0621
2b (Reference)	25 (17.7%)	
Unknown	1 (1%)	
Subglottic Ext		**0.0505**
Yes	18 (12.8%)	
No	96 (68.1%)	
Missing	27 (19.2%)	
Supraglottic Ext		0.4668
Yes	30 (21.2%)	
No	87 (61.7%)	
Missing	24 (17%)	
Median Overall Treatment Time (wks)	6.5 (4–9.7)	
Radiation Dose (cGy) as Continous	6820 (6300–7264)	**0.0367**
≤66 Gy	45	
> 66 Gy	96	
Median Dose/Fraction	212 (176–256)	0.1353
≤2Gy/fx	45 (31.9%)	
>2Gy/fx	95 (67.4%)	
missing	1 (1%)	
Mean Follow-Up (month)	67.7 (0–239)	

**Table 2:** Multivariate Analysis for Impact on Local Control

**Variables**	**p-value **	**HR**	**95% Confidence (LL, UL)**
Heavy Alcohol Use	**0.0006**		
Current vs Never	**0.0002**	42.32	(5.7,309)
Former vs Never	0.4738	0.44	(0.05, 4.2)
Current vs Former	**0.0016**	116.00	(6.0, 1656)
Ant Commisure Inv (n vs y)	0.0940	0.21	(0.03, 1.31)
T Stage	**0.024**		
2b vs 1a	**0.0288**	0.09	(0.01, 0.78)
2b vs 1b	0.2	5.76	(0.4, 83.4)
2b vs 2a	0.8	0.78	(0.12, 5)
Total Dose (continuous variable)	0.246	1.00	(0.99, 1.00)
Field Weighting/Arrangement	**0.0569**		
Bilateral (Weighted) vs Non	**0.0027**	11.36	(1.32, 100)
Bilateral (Weighted) vs Unilateral	**0.0422**	10.70	(1.09, 100)
Bilateral (Non-weighted) vs Unilateral	0.9473	0.93	(0.123, 7.11)
Subglottic Ext (n vs y)	0.7468	0.74	(0.117, 4.860)

### Local control

**Figure 2:**
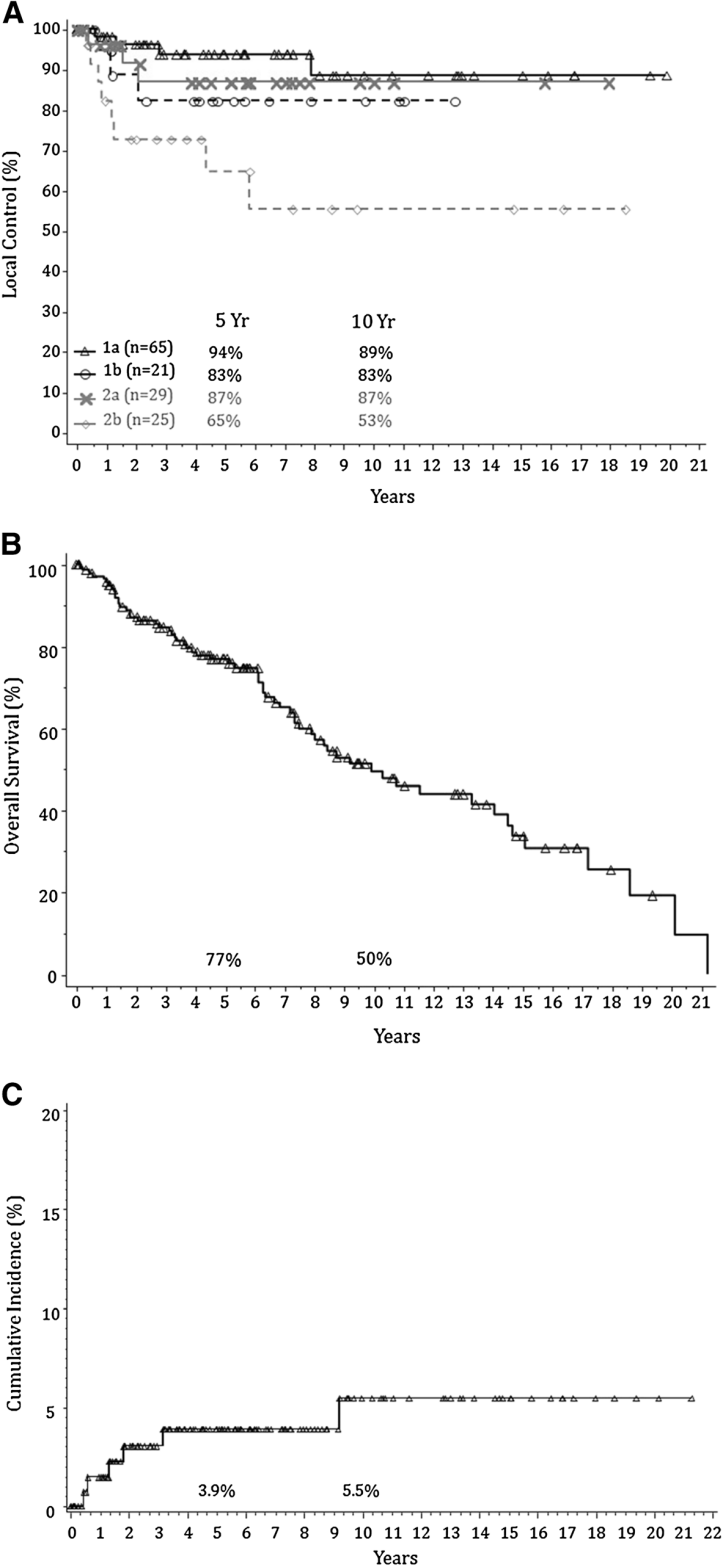
Outcomes Including Local Control Rate (Figure 2A), Overall Survival (Figure 2B), and Cumulative Incidence of Death (Figure 2C) For Early Glottic Carcinoma.

The 5-year actuarial local control rates were as follows: T1a, 94%; T1b, 83%; T2a , 87%; T2b, 65%. The 10-year actuarial local control rates were as follows: T1a, 89%; T1b, 83%; T2a, 87%; T2b, 56% (Figure 2A). A total of 18 patients (12.7%) experienced local failures (Table 3). The most common site of local recurrence was at the initial site of the tumor (6) followed by elsewhere on the true glottis but not at the original site of the primary location (4).

**Table 3:** Outcomes Post Radiation Therapy

**Outcomes Post Radiotherapy**	**n (% of total)**
Local Recurrence	18 (12.8)
Only at Primary Site	6 (4)
Near Primary w/o Primary Site	8 (5.7)
Loco-Regional Recurrenc Including Primary	21 (14.9)
Nodal Failure Only w/o Primary	3 (2.1)
Salvage Surgery	20 (14.2)
Local Control with Salvage Sx	135 (95.7)
Salvage w/o Functional Larynx	11 (7.8)
Metastatic Failure	3 (2.1)
Lower Tracheal/Stomal	1 (0.007)
Lung	1 (0.007)
Other Primary Cancers	32 (22.7)
Most Common Site (Lung)	9 (6.3)

### Neck control, metastatic failures, salvage therapy, and functional larynx

Neck control rates were 97.9% with only 3 (2.1%) of the patients failing in the regional lymph nodes. Similarly, only 3 patients (2.1%) developed non-regional metastatic failure: One in lower trachea and stoma, one in the lung, and one was unknown. The most common form of salvage therapy in 20 patients (14.2%) was total laryngectomy (8), laser cordotomy (6), or subtotal laryngectomy (4). One patient had neither of these procedures and another only had selective lymph node dissection. Six of the patients that were salvaged underwent repeat salvage therapy with either subtotal laryngectomy (1), total laryngectomy (2) or some other procedure (3). The ultimate local control rate of all patients including those that were salvaged (12 out of 18 local failures) with surgery was 95.7%. Of those that were salvaged 11 patients (7.8%) were left without a functional larynx.

### Second tumors, overall survival, and cause specific survival

Thirty-two patients (22.7%) developed second cancers: the most common site was lung (9 patients) and prostate (2 patients). The 5 and 10-year overall survival rates were 77% and 50%, respectively (Figure 2B). The 5 and 10-year cumulative incidence of death were 3.9% and 5.5%, respectively (Figure 2C). A total of 62 deaths (44%) occurred over the study interval. Recurrent larynx cancer as the cause of death occurred in only 5 patients (3.5%). The rest died of non-larynx and non-second cancer related causes (26 patients), other cancers (9 patients), or of unknown causes (22 patients). During the last follow-up, 78 patients (55.3%) were alive without recurrent disease or were alive with recurrent disease (5 patients, 3.5%).

### Voice quality and toxicity outcomes

No patients experienced severe or fatal post radiation complications. Pre and post-treatment symptoms are shown in Table 4. 92% were noted to have baseline impairment of voice prior to radiation. 73% of these patients reported significant improvement, while 8.5% noted chronic worsening of their voice. Chronic laryngeal edema was noted in 26 patients (18.4%). Chronic dysphagia was noted in 9 patients (6.4%). Only one patient (0.7%) developed severe laryngeal edema requiring a tracheostomy (grade 4), and only one patient (0.7%) developed severe dysphagia requiring long term tube feeding (grade 4).

**Table 4:** Pre and Post Treatment Quality of Life

			**Number**	**(%)**
**Pre-Treatment**				
	Voice Quality			
		Impaired	129	91.5
		Normal	4	2.8
		missing	8	5.7
	Trismus (pretx)			
		N	123	87.2
		Y	2	1.4
		missing	16	11.3
**Post Treatment**				
	Chronic Hoarseness			
		Improved	103	73.0
		Unchanged	11	7.8
		Worsened	12	8.5
		missing	15	10.6
	Trismus (posttx)			
		Improved	1	0.7
		None	126	89.4
		missing	14	9.9
	Chronic Laryngeal Edema			
		None	101	71.6
		Mild/Mod	24	17.0
		Severe	1	0.7
		Very Severe	1	0.7
		missing	14	9.9
	Chronic Dysphagia			
		None	118	83.7
		Mild	7	5.0
		Moderate	1	0.7
		Severe	1	0.7
		missing	14	9.9

## Discusion

Our observed five-year LC for T1-T2a are excellent and are better than or comparable to others [10]. The slight differences in LC could be due to the fact that we included all patients in our study, including three patients with spindle cell variant histology. All three of these patients failed locally suggesting the need for more aggressive treatment in these patients. Another reason could be due to the fact that we used a lower daily fractional dose and avoided hyper-fractionationation. Despite these subtle differences, we did not find total dose or the fractional dose to be statistically significant on MVA. This finding taken together with excellent outcome for T1a patients suggests that current treatment policies could be improved by reducing the total radiation dose or avoiding hyper-fractionation in these patients.

The relatively poor outcomes of patients with T2b glottic carcinoma suggests that there are various degrees of impaired mobility which are not adequately assessed with existing methods. The impaired mobility is an indication of the extent of disease. Some of these patients maybe on the verge of being T3 lesions, which would suggest the need for more aggressive treatment strategies such as hyper-fractionation or the use of concurrent chemo-radiotherapy. Trotti [13] et al. reported a non-statistically significant improvement of 9% in LC at five years (p= 0.11) for the use of hyper-fractionation in T2N0 glottic cancer patients. Akimoto [14] et al. reported that concurrent chemoradiotherapy had a non-significant improvement in disease free survival of 91.8% at 5 years compared to 70.9% no chemotherapy. Nonoshita et al. [15] reported an impressive three-year local control of 95.4% with the use of concurrent tegafur. Another treatment approach may involve some sort of limited surgical resection combined with neoadjuvant or adjuvant chemoradiotherapy. This approach has yet to be tested in a randomized trial.

Our analysis identified tumor stage (especially T2b), concurrent heavy alcohol consumption, and field weighting as factors that adversely impacted local control. Anterior commissure involvement was also noted to be statistically significant on MVA when we compared T2 patients with T1 patients. Tumor stage [3,7,9,10] and anterior commissure[3,8] involvement have also been reported by others to predict for poor local control. Other factors previously reported by others to poorly impact local control on multivariate analysis include: total dose (<65 Gy)[8]; overall treatment time[5,8] (> 41 days[10]); poorly differentiated histology[10]; smaller fraction sizes[5,8,9] (< 2Gy vs > 2 Gy[6]); subglottic extension[6-8]; treatment delay (> 3 days)[2]; treatment interruptions[2]; age, smaller field sizes[2,9]; gender[3,7]; higher beam energy[3]; pre-treatment hemoglobin levels[4,7]; impaired vocal cord mobility[3,4,8]; and larger tumor extent[3] (or tumor bulk[7]). We did not find these factors to be statistically significant and could be due to the fact that our patients were treated in a very homogeneous manner. Most patients were treated to a dose above 66 Gy, overall treatment time was kept short, larger fraction sizes were used, and there were minimum treatment delays and interruptions. Furthermore, most patients were treated with 6 MV energy beams. It is unclear why heavy alcohol consumption during radiation was significant for local control. One reason could be due to the multi-organ effect of alcohol on the immune system and the bone marrow system during radiation. Another reason could be due to unknown biological mechanisms leading to reduced radiation damage within the cancer cells at the DNA level allowing cancer cells to become more radioresistant. Future work should address why concurrent heavy alcohol impacts local control in prospective studies.

Nearly all of our patients had baseline voice impairment with 73% noting significant improvement and 8.5% reported worsening after radiotherapy. Radiotherapy is considered an acceptable treatment option and has similar [16] to slightly superior [17] patient reported voice quality outcomes compared to CO2 laser excision, especially for T1 glottic carcinoma patients. However, radiotherapy is more costly [17] and provides no additional benefit in terms of differences in local control, laryngectomy free survival, or overall survival compared to C02 laser excision [17]. Radiation may be an option for those unwilling to undergo surgery or for those with multiply recurrent lesions after translaser oral excision. Future health policies will need to address cost-effective care for such patients through well controlled randomized trials. Patients with T2 disease often require more extensive surgical excision, thus the voice quality is better with radiotherapy and open partial larygenctomy may be more expensive [10].

There are several limitations. For one, our study is a single institutional retrospective review spanning over three decades. A direct comparison of radiotherapy with alternative treatment strategies such as transoral CO2 laser excision or an open partial laryngectomy is better made via a randomized controlled clinical trial. Another limitation includes availability of only subjective measures of patient reported and physician reported voice quality measures. Lastly, tumor volume and tumor volume dosimetry is better captured in modern 3D treatment techniques.

## Conclusion

Definitive radiotherapy provides excellent LC and CSS for T1-T2a, N0 glottic SCC, with excellent voice preservation outcomes and minimal long term toxicity. T2b tumors have inferior outcomes, and alternative management strategies should be pursued.

## Competing interest

The authors declare that they have no competing interests.

## Authors’ contribution

MKK, SAK, and GKH participated in the study design and coordination, performed acquisition of data, and drafted the manuscript. CPR participated in the statistical data analysis. JPS was the treating physician over the 20 years and provided technical oversight to the project. All authors reviewed and approved the final manuscript. Our study was presented in an abstract form at the annual meeting of the American Society of Therapeutic Radiation Oncology in 2009 [18].
